# Retraction: The impact of online learning community participation on college students' psychological capital: the mediating role of academic identity and the moderating role of psychological resource transformation

**DOI:** 10.3389/fpubh.2026.1901134

**Published:** 2026-06-09

**Authors:** Frontiers Editorial Office

The journal retracts the 09 March 2026 article cited above.

Following publication, concerns were raised regarding the validity of the data in the article. The authors failed to provide a satisfactory explanation during the investigation, which was conducted in accordance with Frontiers' policies.

This retraction was approved by the Chief Editors of Frontiers in Public Health and the Chief Executive Editor of Frontiers. The authors received a communication regarding the retraction and had a chance to respond. This communication has been recorded by the publisher.

